# Immunohistochemical Expression of SGLT2 in Pancreatic Ductal Adenocarcinoma

**DOI:** 10.7759/cureus.89878

**Published:** 2025-08-12

**Authors:** Shristi Butta, Manoj Gupta

**Affiliations:** 1 Pathology/Oncopathology, Institute of Post Graduate Medical Education and Research (IPGMER) and Seth Sukhlal Karnani Memorial Hospital (SSKM) Hospital, Kolkata, IND; 2 Tropical Medicine, Calcutta School of Tropical Medicine, Kolkata, IND

**Keywords:** ductal pancreatic carcinoma, histopathological grades, immunohistochemistry (ihc), sglt2 gene, tumor stage

## Abstract

Background: Pancreatic ductal adenocarcinoma (PDAC) is an aggressive neoplasm. Sodium-glucose transport protein 2 (SGLT2) is a sodium-dependent glucose transporter involved in glucose reabsorption from the kidney. This provides the energy required for the viability of the cells. In this study, we aim to determine the expression of SGLT2 by immunohistochemistry (IHC) in various histological grades and tumor stages of PDAC.

Materials and methods: This retrospective study was conducted in a tertiary care center and included 41 cases of PDAC. The study duration was 18 months. The paraffin blocks were obtained from archives and histopathologically processed. IHC was done using rabbit polyclonal anti-SGLT2 antibody.

Results: Males were more frequently affected (n=34; 82.93%) as compared to females (n=7; 17.07%); 43.9% (n=18) of the cases were aged >50 years; 70.73% (n=29) of the cases were histologically graded as moderately differentiated adenocarcinoma; 73.17% (n=30) of the cases revealed a positive history of impaired glucose tolerance or diabetes mellitus; this was found to be significantly associated with the histological grade of PDAC (p=0.0053); 65.85% (n=27) of the cases were staged as pT2; 58.54% (n=24) of the cases showed a moderate expression of SGLT2, while 9.76% (n=4) of the cases showed a strong expression. A statistically significant association was found between histological grade and SGLT2 immunoexpression (p=0.0001). A significant association was also found between SGLT2 immunoexpression and pT (tumors) stage (p=0.0002).

Conclusion: SGLT2 immunoexpression is significantly associated with the histological grade and tumor stage of PDAC. Hence, we suggest that SGLT2 inhibition can be a potential therapeutic strategy in moderate to advanced PDAC.

## Introduction

Pancreatic ductal adenocarcinoma (PDAC) is an aggressive neoplasm. It is the fourth leading cause of cancer-related mortality [1]. This histologic subtype of pancreatic cancer accounts for greater than 90% of all pancreatic cancer cases. The five-year survival rate is reported to be as low as 7% [2-4]. Frank symptoms are rarely seen, and hence, diagnosis is delayed. The main modality of treatment includes the Whipple procedure (pancreatoduodenectomy), which is a radical procedure with several limitations and contraindications, like distant metastasis, tumors involving the superior mesenteric artery, celiac trunk, inferior vena cava, aorta, and tumors resulting in superior mesenteric vein occlusion [5]. Further, the Whipple procedure is the surgical procedure of choice only for the resectable and borderline resectable PDACs. Hence, there is a necessity to identify a molecule that could act as a marker for disease progression and as a potential therapeutic target, especially in moderate to advanced PDAC, in combination with chemotherapy.

About 90% of PDACs are associated with a KRAS mutation. The most significant metabolic alteration driven by the KRAS mutation is glycolysis. This glycolysis is important in epithelial-mesenchymal transition (EMT), tumor angiogenesis, and distant metastasis in PDAC [6]. The phenomenon of aerobic glycolysis is well documented as the Warburg effect, which suggests that cancer cells uptake glucose and ferment it to lactate to generate energy, even in the presence of oxygen [7].

There are two main types of glucose transport proteins in humans. One group includes the glucose transporter proteins (GLUTs), and the other group includes the sodium-glucose transport proteins (SGLTs). GLUTs are responsible for facilitated diffusion of glucose, while SGLTs are responsible for sodium-dependent glucose transport. SGLT2 is located in the proximal renal tubule. Around 80% to 90% of the glucose is reabsorbed by SGLT2, while the remainder is reabsorbed by SGLT1. SGLT2 inhibitors, or gliflozins, are a class of established antidiabetic drugs. The gliflozins include canagliflozin, dapagliflozin, and empagliflozin. By inhibiting SGLT2, gliflozins prevent the reuptake of glucose from the glomerular filtrate and further lower the glucose level in the blood and promote the excretion of glucose in urine. These drugs do not alter the insulin levels and hence can be given to non-diabetics as well.

Several tumors, like hepatocellular carcinoma, breast carcinoma, pancreatic carcinoma, prostatic carcinoma, lung and colonic carcinoma, and glioblastoma, have shown SGLT expression (SGLT1 or 2).

However, to the best of our knowledge, no study has to date demonstrated any association between the SGLT2 immunoexpression and the pT (tumors) stage and histological grade of PDAC. We would hypothesize that a higher expression of SGLT2 could be associated with advanced tumor grade and stage, thereby supporting the rationale of exploring the potential role of SGLT2 inhibition in PDAC in future studies. 

Here in this study, we aim to determine the immunohistochemical expression of SGLT2 in PDAC and to establish the association between SGLT2 and various histological grades and pT stages of PDAC.

## Materials and methods

This retrospective study, which spanned a period of 18 months, was conducted in the department of pathology of the Institute of Post Graduate Medical Education and Research (IPGMER) and Seth Sukhlal Karnani Memorial Hospital (SSKM), a tertiary care hospital in Kolkata, West Bengal, India. The study included 41 cases of PDAC that had undergone a radical Whipple procedure with the following inclusion criteria: 1. Cases that were histologically confirmed as PDAC; 2. Cases that underwent pancreatoduodenectomy (Whipple's procedure); 3. Cases with complete availability of clinicopathological data were included in the study; 4. Cases with adequate tissue on paraffin blocks obtained from the archives. 

Exclusion criteria included non-PDAC cases, previously treated cases, as this could alter the SGLT2 immunoexpression, inadequate tissue on the paraffin blocks, recurrent cases, and non-Whipple's resection cases. 

The formalin-fixed paraffin-embedded (FFPE) blocks were obtained from the archives and sent for histopathological and immunohistochemical analysis. Five-micrometer-thick sections were cut and stained with routine hematoxylin and eosin (H&E) stain. Only cases microscopically diagnosed as PDAC were included in the study. Pathological tumor, node, metastasis (pTNM) staging was done using the American Joint Committee on Cancer (AJCC) 8th Edition staging manual [8].

Immunohistochemistry (IHC) was performed using rabbit polyclonal SGLT2 antibody in the following manner. Three-micrometer sections of FFPE blocks were cut for IHC. Heat-induced epitope retrieval (HIER) of the antigen was done using a pressure cooker, with Tris ethylenediaminetetraacetic acid (EDTA) buffer at a pH of 9.0. The endogenous peroxidase activity was counteracted by a drop of 3% hydrogen peroxide and incubating it for 10 minutes. Further, the sections were incubated with rabbit polyclonal SGLT2 antibody for 60 minutes. Subsequently, sections were treated with horseradish peroxidase (HRP) label, which was used as a secondary antibody, and 3,3'-diaminobenzidine (DAB) chromogen was applied. The counterstain used was hematoxylin.

SGLT2 was considered positive when malignant ducts showed immunopositivity in tumor hotspots. Normal kidney cortex was used as a positive control. Cytoplasmic and membrane positivity for SGLT2 was considered positive. Depending on the intensity, SGLT2 IHC was scored as weak, moderate, or strong.

Statistical analysis was performed using the Epi Info^TM^ software version 7.2.6.0 (CDC’s Center for Surveillance, Epidemiology, and Laboratory Services (CSELS), Atlanta, GA). A p-value of less than 0.05 was considered significant. 

## Results

Males were more frequently affected, accounting for 82.93% (n=34) of the cases, while 17.07% (n=7) of cases were seen in females; 43.9% (n=18) of the cases were aged between 50 and 70 years. Pain was the chief complaint in 68.29% (n=28) of the cases, followed by other constitutional symptoms in 31.71% (n=13) of the cases. Smoking was associated with 39.02% (n=16) of the cases. Alcohol consumption was associated with 51.22% (n=21) of the cases. However, alcohol consumption was not significantly associated with histological grade (p=0.5765) or SGLT2 immunoexpression (p=0.3108); 73.17% (n=30) of the cases revealed a positive history of impaired glucose tolerance or diabetes mellitus. 

**Figure 1:**
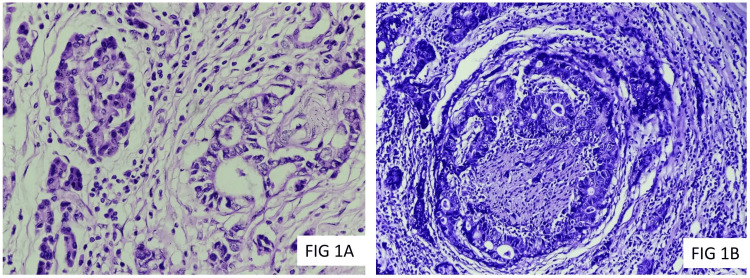
1A: Moderately differentiated pancreatic ductal adenocarcinoma (H&E, 200X); 1B: Perineural invasion in pancreatic ductal adenocarcinoma (H&E, 100X)

**Figure 2:**
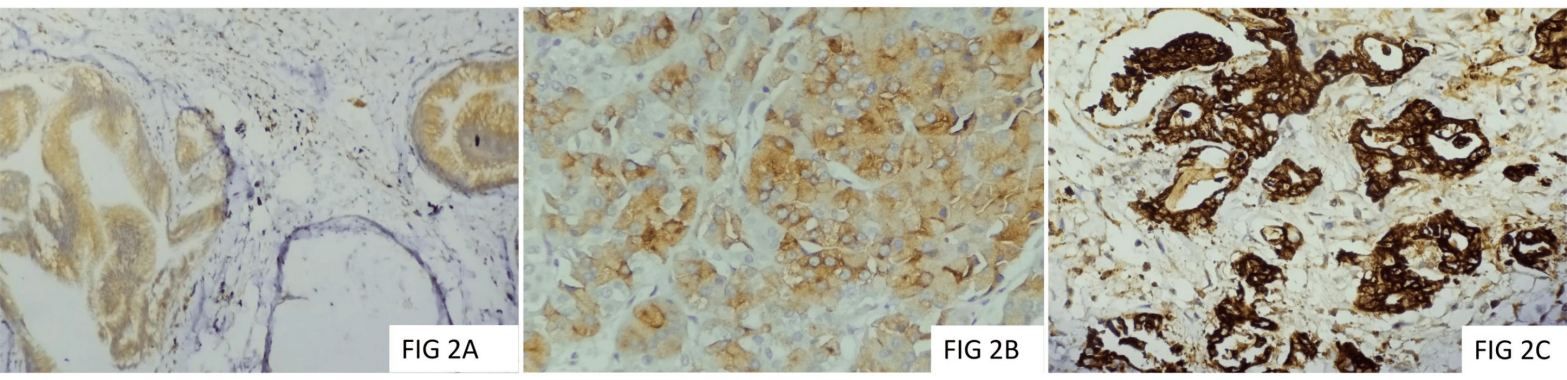
Cytoplasmic and membrane SGLT2 immunoexpression (2A: weak; 2B: moderate; 2C: strong) (SGLT2, 200X)

Among the study group, 70.73% (n=29) of the cases were histologically graded as moderately differentiated adenocarcinoma (Grade 2), while 26.83% (n=11) were well-differentiated adenocarcinoma (Grade 1), and 2.44% (n=1) of the cases were graded as poorly differentiated adenocarcinoma (Grade 3). Histological grade was found to be significantly associated with a history of impaired glucose tolerance or diabetes mellitus (p=0.0053). The majority of the cases (n=27; 65.85%) were staged as pT2, 17.07% (n=7) of the cases were staged as pT1, and pT3. None of the cases were staged as pT4. Nodal metastasis (pN1) was noted in 29.27% (n=12) of the cases. None of the cases revealed pN2 nodal stage. Distant metastasis was not reported in any of the cases. 58.54% (n=24) of the cases showed a moderate expression of SGLT2, while 9.76% (n=4) of the cases showed a strong immunoexpression, whereas 24.39% (n=10) of the cases revealed weak immunoexpression for SGLT2 (Figures 1-2).

Significant association was found between histological grade and SGLT2 immunoexpression (p=0.0001). A significant association was also found between SGLT2 immunoexpression and pT stage (p=0.0002) (Tables 1-3). However, no significant association was found between SGLT2 immunoexpression and pN stage(p=0.1992).

**Table 1:** The association between impaired glucose tolerance or diabetes mellitus with histologic grade (p=0.0053) A p-value of <0.05 is considered statistically significant. The data have been represented as the number of cases (N); chi-square: 10.43; degree of freedom (df): 2; p-value: 0.0053

Parameters	Histologic grade			
Associated impaired glucose tolerance or diabetes mellitus	Number of moderately differentiated adenocarcinoma cases	Number of poorly differentiated adenocarcinoma cases	Number of well-differentiated adenocarcinoma cases	Total number of cases
No	4 (13.79%)	0 (0.00%)	7 (63.64%)	11 (26.83%)
Yes	25(86.21%)	1 (100.00%)	4 (36.36%)	30 (73.17%)
Total	29 (100%)	1 (100%)	11 (100%)	41 (100%)

**Table 2:** The association between histologic grade and SGLT2 immunoexpression (p=0.0001) p<0.05 is considered statistically significant. The data have been represented as the number of cases (N); chi-square: 28.4258; degree of freedom (df): 6; p-value: 0.0001; IHC: immunohistochemical

Histologic Grade	SGLT2 IHC expression				
	Moderate	Negative	Strong	Weak	Total
Number of moderately differentiated adenocarcinoma cases	22 (91.67%)	0 (0.00%)	3 (75%)	4 (40%)	29 (70.73%)
Number of poorly differentiated adenocarcinoma cases	0 (0.00%)	0 (0.00%)	1 (25%)	0 (0.00%)	1 (2.44%)
Number of well-differentiated adenocarcinoma cases	2 (8.33%)	3 (100.00%)	0 (0.00%)	6 (60%)	11 (26.83%)
Total number of cases	24 (100%)	3 (100%)	4 (100%)	10 (100%)	41 (100%)

**Table 3:** The association between SGLT2 immunoexpression and pT stage of the tumor (p=0.0002) p<0.05 is considered statistically significant; The data have been represented as the number of cases (N); chi-square: 26.5126; degree of freedom  (df): 6; p-value: 0.0002; pT stage: pathological tumor stage

SGLT2 IHC expression	pT stage			
	Number of cases with pT1 stage(<=2cm)	Number of cases with pT2 stage(>2-4cm)	Number of cases with pT3 stage (>4cm)	Total number of cases
Moderate	2 (28.57%)	19 (70.37%)	3 (42.86%)	24 (58.54%)
Negative	3 (42.86%)	0 (0.00%)	0 (0.00%)	3 (7.32%)
Strong	0 (0.00%)	1 (3.70%)	3 (42.86%)	4 (9.76%)
Weak	2 (28.57%)	7 (25.92%)	1 (14.28%)	10 (24.39%)
Total	7 (100%)	27 (100%)	7 (100%)	41 (100%)

## Discussion

PDAC is a highly aggressive neoplasm with an adverse prognosis. Despite the several traditional treatment modalities available, like surgery, chemotherapy, and radiation, the tumor has a five-year survival rate of less than 10% [9]. This necessitates the need to open newer avenues for ascertaining the tumor progression and to identify potential therapeutic strategies for better patient outcomes.

This study found that SGLT2 immunoexpression was significantly associated with both the tumor stage (pT) and histological grade in patients with PDAC. Well-differentiated adenocarcinoma mainly showed a weak expression of SGLT2, while moderate-to-poorly differentiated cases revealed a moderate to strong immunoexpression for SGLT2. The majority of the pT2 tumors showed a moderate immunoexpression for SGLT2. The above findings indicate that SGLT2 immunoexpression is strongly associated with tumor progression in PDAC. Similar observations were also made by Ren et al., who found that SGLT2 promoted PDAC progression by activating the Hippo signaling via the hnRNPK-YAP1 axis [10]. Further, there is a reasonable difference between glucose transport mediated by GLUT channels and by SGLT2 channels. GLUT channels in healthy individuals drive glucose along the concentration gradient to maintain the functional activity of healthy cells. On the other hand, SGLTs use the sodium gradient to allow the uptake of glucose. It has been hypothesized that SGLTs have the ability to allow for glucose uptake for cellular metabolism even when the glucose concentration in the tumor microenvironment is quite low. The expression of SGLT2, besides the pancreas, has also been documented in liver, prostate, bowel, lung, and breast cancers [11]. The cancerous cells, besides expressing GLUT, also express SGLT2. However, there is no data regarding the proportion of the two channels in tumor cells. Attempts to target GLUT channels have not been quite successful in the past, owing to the expression of GLUTs in healthy cells as well. However, selective SGLT2 inhibitors can serve as an important therapeutic strategy because, besides the cancer cells, these are often expressed in the proximal renal tubules only.

Scafoglio et al. found that SGLT2 inhibition could result in tumor necrosis and decreased growth in pancreatic cancers in mouse models [12]. SGLT2 drives glucose into areas of tumors that are oxygen-deficient. These hypoxic areas in the tumor are usually resistant to the chemotherapeutic agents [13]. Hence, this suggests that SGLT2 inhibitors could be combined with the chemotherapeutic agents to enhance the anti-proliferative effect in PDAC.

Regarding the epidemiological parameters, we found that males were more prone to develop PDAC as compared to females. This could possibly be due to the increased exposure of known risk factors like alcohol intake and smoking among males as compared to females. These findings have corroborated with the global data as well [14]. The majority of the patients in our study belonged to the fifth to seventh decades of life. These findings were quite similar to those given by Bray et al. [15]. The reason for this late onset can possibly be ascribed to the genetic alterations that accumulate over time, resulting finally in the development of a malignant neoplasm. The majority of our cases showed an impaired glucose tolerance, and some even showed diabetes mellitus. In a review of literature by Khadka et al., it was found that about 85% of pancreatic cancer cases were associated with impaired glucose tolerance and some even with diabetes mellitus [16]. Further, diabetes was found to improve with pancreatic resection. Further, several studies from the past have found a positive association between diabetes mellitus and pancreatic cancer [17-19]. This could be explained by altered glucose metabolism in patients of PDAC with KRAS mutation, which is the most common mutation, seen in about 90% of the cases [20]. Further, diabetes mellitus is associated with a 1.8 times increased risk of pancreatic cancers in Asian and Hispanic men as compared to White and Black men. [21-22] This suggests that an impaired glucose tolerance or diabetes mellitus could be considered a precancerous condition for PDAC. Hence, new-onset impaired glucose tolerance could serve as an early manifestation of an occult PDAC. We also found that a significant association existed between patients with impaired glucose tolerance or diabetes mellitus and the histological grade of the tumor. As our sample size was small and because of the growing burden of diabetes mellitus in India, establishing this association requires more such studies.

The majority of our cases revealed a moderate to strong cytoplasmic expression of SGLT2. This was found to have a significant association with tumor histological grade, indicating that SGLT2 could act as a potential biomarker for moderate to advanced PDAC. In addition, SGLT2 was also found to be significantly associated with the pT stage of the tumor. Kobayashi et al. studied the SGLT2 expression by IHC in clear cell renal cell carcinoma and found that increased expression of SGLT2 has been associated with a poor prognosis and a decreased overall survival [23]. Further, Wright et al. studied the expression of SGLT2 in pancreatic cancers, prostate cancers, and glioblastomas and highlighted that Me4FDG could act as a new radiotracer to detect SGLT activity, unlike 2FDG, which acts as a substrate for GLUT1 in FDG-PET scans [24]. Hence, SGLT2 IHC, if done in all cases of PDAC, can aid in ascertaining the aggressive potential of the tumor. In addition, we would also like to suggest that SGLT2 inhibitors could act as potential anti-proliferative agents in such cases.

However, this exploratory study has several limitations, too. A retrospective single-center study restricts its generalizability and selection bias. Additionally, tumor heterogeneity could contribute to varying levels of immunoexpression of SGLT2 within the same tumor. Further, SGLT2 being a metabolically active protein, additional radio-imaging techniques could be employed to ascertain metabolically active zones in the tumor. Hence, future studies could help provide better insights into the tumor biology by integrating imaging with IHC in metabolically active zones of the tumor. Further, integrating molecular assays with IHC can better validate the results of the study. Furthermore, as no follow-up data on survival were available, the prognostic utility remains to be validated in future studies. Additionally, normal kidney cortex was used as a positive control. However, a normal pancreas or pancreatitis can also be used as a positive control in future studies to create a standard for measuring the overexpression of SGLT2. Moreover, only semi-quantitative scoring was used, which can lead to inter-observer variability. Hence, using standardized scoring techniques with image-based analysis can be one of the future endeavors.

## Conclusions

SGLT2 is mainly associated with glucose reabsorption; however, it also holds a potential role in the tumor growth, survival, and chemotherapeutic resistance in PDAC. IHC for SGLT2 could serve as a potent adjunct in ascertaining the tumor prognosis. Higher immunoexpression might be associated with poor prognosis. Additionally, SGLT2 inhibitors could potentially serve as an adjunct to the usual therapeutic options in PDAC.
